# Cleavage Stimulation Factor Subunit 2: Function Across Cancers and Potential Target for Chemotherapeutic Drugs

**DOI:** 10.3389/fphar.2022.852469

**Published:** 2022-03-18

**Authors:** Linfei Feng, Fengyang Jing, Xiaofeng Qin, Liming Zhou, Yujie Ning, Jun Hou, Weihao Kong, Youming Zhu

**Affiliations:** ^1^ Department of Oral and Maxillofacial Surgery, The First Affiliated Hospital of Anhui Medical University, Hefei, China; ^2^ Key Laboratory of Oral Diseases Research of Anhui Province, Department of Dental Implant Center, Stomatologic Hospital and College, Anhui Medical University, Hefei, China; ^3^ Department of Emergency Surgery, Department of Emergency Medicine, The First Affiliated Hospital of Anhui Medical University, Hefei, China

**Keywords:** cleavage stimulation factor subunit 2, pan-cancer, biomarker, drug targets, bioinformatics

## Abstract

The cleavage stimulation factor subunit complex is involved in the cleavage and polyadenylation of 3′-end pre-mRNAs that regulate mRNA formation and processing. However, cleavage stimulation factor subunit 2 (*CSTF2*) was found to play a more critical regulatory role across cancers. General cancer data sets from The Cancer Genome Atlas and Genotype-Tissue Expression project were thus downloaded for differential analysis, and the possible functions and mechanisms of CSTF2 in general cancer were analyzed using the Compartments database, cBioPortal database, Tumor Immune Single-cell Hub database, and Comparative Toxigenomics database using gene set enrichment analysis and R software. The results showed that *CSTF2* could affect DNA repair and methylation in tumor cells. In addition, CSTF2 was associated with multiple tumor immune infiltrates in a wide range of cancers, and its high expression was associated with multiple immune checkpoints; therefore, it could serve as a potential target for many drug molecules. We also proved that CSTF2 promotes oral cell proliferation and migration. The high diagnostic efficacy of *CSTF2* suggested that this gene may act as a new biomarker and personalized therapeutic target for a variety of tumors.

## 1 Introduction

The cleavage stimulation factor subunit 2 (CSTF2), also known as CSTF-64, is one of three subunits of the CSTF complex, the other two being CSTF-50 (gene symbol *CSTF1*) and CSTF-77 (gene symbol *CSTF3*) (Grozdanov et al., 2018a). CSTF2 is one of the first proteins identified in the 3′-terminal processing complex of pre-mRNA based on strong and specific UV crosslinking of CSTF2 with RNA containing A functional poly (A) signal (Liu et al., 2007; Tardif et al., 2010; Gruber et al., 2016).

CSTF2 encodes a 557-amino acid protein that contains a conserved RNA recognition motif (RRM) RNA binding domain at its N-terminus, a long proline region rich in glycine, and a pentapeptide repeat region that forms an extended alpha-helix (Steber et al., 2019). In the RRM of CSTF2, there is a U dinucleotide specific binding site and a highly mobilized protein: through its RRM, CSTF2 binds to the U or GU rich sequence downstream of the cleavage site to form a stable complex, affecting the processing of pre-mRNA (Grozdanov et al., 2020). Therefore, CSTF2 is essential for the cleavage of mRNA and polyadenosine (C/P).

Studies have shown that the expression level of CSTF2 is positively correlated with the shortening trend of global mRNA 3′-UTR. The deletion of miRNA complementary sites in some 3′-UTR of oncogenes is one of the mechanisms of oncogene activation. In cancer cells, the 3′-UTR of some oncogenes may be shortened by alternating cleavage and polyadenylation, thereby avoiding miRNA silencing and oncogene activation in cancer cells (Liu et al., 2007; Youngblood et al., 2014; Barra et al., 2020). CSTF2 may play an important role in the activation of some oncogenes by altering the 3′-UTR length of oncogenes in cancer cells.

Recent studies have shown that CSTF2 is also important as a regulator of alternate polyadenylate (APA). *CSTF2* is often trans-activated in most primary lung cancers and is often overexpressed in clinical lung cancer specimens and cell lines (Kargapolova et al., 2017; Sudheesh et al., 2019). Expression of CSTF2 is associated with shortening of the 3′-UTR of differentially expressed genes in lung cancer. The gene may play an indispensable role in the growth and invasion of lung cancer cells (Aragaki et al., 2011; Delfanti et al., 2021).

However, there are few reports on the regulatory role and function of CSTF2 in pan-cancer. As a key regulatory molecule in the nucleus, the regulatory function of CSTF2 in many cancers is still unknown. Therefore, in our study, the effects of CSTF2 on DNA mismatch repair and methylation in various cancer tumors were analyzed *via* a pan-cancer analysis, and on immune infiltration in the tumor microenvironment, as well as its value as an immune checkpoint. The diagnostic and prognostic value of CSTF2 in a pan-cancer analysis was also evaluated in hopes to find new diagnostic biomarker and target for the treatment of tumors.

## 2 Materials and Methods

### 2.1 Gene Expression Analysis

Pan-cancer sequencing data (Illumina platform), including 10,363 tumor samples from The Cancer Genome Atlas (TCGA) database and 5,413 normal samples from the Genotype-Tissue Expression (GTEx) database, as well as data linked to the Human Protein Atlas (www.proteinatlas.org/) database were extracted through their portal websites for analysis (Tomczak et al., 2015). The whole data collection was filtered, removing missing and duplicated results, and transformed by log2 (TPM +1), using the rma function within the R package. The R package ggplot2 was used for visualization results.

We used the Compartments database (http://compartments.jensenlab.org) to predict the subcellular localization of CSTF2, then searched the cBio Cancer Genomics Portal (cBioPortal, http://www.cbioportal.org/) database and analyzed the Pan-Cancer Atlas dataset based on TCGA (Cerami et al., 2012; Gao et al., 2013), which included CSTF2 mutations in 10, 967 samples.

### 2.2 Functional Enrichment Analysis of Genes

We used the Gene Expression Profiling Interactive Analysis (GEPIA) database (http://gepia.cancer-pku.cn/) (Tang et al., 2017) to analyze similar genes for CSTF2. To analyze the relationships between these genes, we used STRING (Szklarczyk et al., 2019) database (https://string-db.org/), which generated a network image using a spring model, with nodes modeled as masses and edges modeled as springs. We then used Cytoscape software (version 3.7.2) to visualize the results and look for hub genes.

### 2.3 Immune Cell Infiltration and Enrichment

Tumor Immune Estimation Resource (TIMER, http://timer.cistrome.org) is a database-based web tool for computing the infiltration of immune cells, which provides infiltration scores for six common immune cells, including B cells, CD4+ T cells, CD8+ T cells, macrophages, neutrophils, and dendritic cells (Li et al., 2016; Li et al., 2017). Immune cell infiltration score of pan-cancer data in TCGA database was calculated by using TIMER software and filed online. Here, we downloaded the infiltrating data and used it to detect correlations expressed by *CSTF2*. We analyzed *CSTF2* expression in various immune cells at the single-cell level using Pan-Cancer datasets in The Tumor Immune Single-cell Hub (TISCH, http://tisch.comp-genomics.org/) database (Sun et al., 2020).

### 2.4 Drug Target Prediction

We analyzed the correlation between CSTF2 and the IC_50_ of drugs by Genomics of Drug Sensitivity in Cancer database (Yang et al., 2013). For drug, disease, and target prediction, we used the Comparative Toxigenomics database (CTD, https://ctdbase.org) (Davis et al., 2021), which provides manually curated information about chemical–gene/protein interactions, chemical–disease and gene–disease relationships. These data were integrated with functional and pathway data to aid in development of hypotheses about the mechanisms underlying environmentally influenced diseases.

### 2.5 Gene-Drug Interactions

The drug-gene interaction database (DGIdb) was used to analyze the interaction between genes and drugs (Sharon et al., 2021). Using a combination of expert curation and text-mining, drug-gene interactions have been mined from DrugBank, PharmGKB, Chembl, Drug Target Commons, and TTD, among others.

### 2.6 Diagnosis and Prognosis Analysis

In this study, the survival data of TCGA-derived pan-cancer patients were statistically analyzed using the R package Proc and Survival, and the analysis results were visualized using the R package ggplot2, and SurvMiner, respectively. RNASeq data in fragments per kilobase per million (FPKM) format were converted into TPM (transcripts per million reads) format and analyzed after grouping according to molecular expression (Liu et al., 2018).

### 2.7 Cell Culture

The HN6 and HEK 293T cell lines were bought from the American Type Culture Collection and maintained in the suggested media and incubated at 37°C in a humidified atmosphere with 95% air and 5% CO_2_.

### 2.8 RNA Interference

For CSTF2 inhibition in HN6 cells, the specific plko.1-shCSTF2 plasmid was transfected into the cells with lipofectamine 3000 (Invitrogen) according to the manufacturer’s instructions, while the plko.1 plasmid was used in the control group. The plko.1-shCSTF2 plasmid was obtained from the Division of Life Sciences and Medicine, University of Science and Technology of China. The shCSTF2 sequence was 5′-CCG​GGC​GTC​TGT​TCA​CTT​TAA​GTT​ACT​CGA​GTA​ACT​TAA​AGT​GAA​CAG​ACG​CTT​TTT​TG-3′.

### 2.9 Quantitative Real-Time PCR Assay

For quantitative real-time PCR (qRT-PCR) analysis, total RNA was extracted using TRIzol (Sigma-Aldrich, United States). cDNA synthesis was performed using a PrimeScript RT kit following the manufacturer’s instructions (Takara, Japan). qRT-PCR was performed as a 20-µL (2 × PCR Master Mix 10 μL, Fwd Primer 1 μL, Rev Primer 1 μL, cDNA 2 μL, nuclease-free water 6 µL) reaction using SYBR premix Ex Taq (Takara, Japan), and the results were analyzed using a Stratagene Mx3000p system (Agilent Technologies, United States). Quantitative PCR was performed under the following conditions: 1) initial denaturation at 94°C for 30°s, 1 cycle; 2) denaturation at 94°C for 30°s, followed by annealing at 58°C for 30°s and extension at 72°C for 30°s, 40 cycles; 3) final extension at 72°C for 7°min, 1 cycle. A relative comparison method was used for qPCR analysis. The β-Actin and CSTF2 primers were 5′-CTG​GAA​CGG​TGA​AGG​TGA​CA-3′ and 5′-AAG​GGA​CTT​CCT​GTA​ACA​ATG​CA-3′ or 5′-TTT​CTC​GGA​GGT​TGG​TTC​TGT​TGT​C-3′ and 5′-ATT​GAG​GTT​CCG​CAT​GGC​ACT​AAG-3′, respectively.

### 2.10 Cell Proliferation and Migration

The HN6 cells of the control group and the shCSTF2 were inoculated into 12-well plates (1 × 10^5^ cells/well), and the digestive counts of the two groups were performed on days 1, 2, and 3. The proliferation curves were plotted and statistically analyzed. The scratch assay was used to measure cell migration. Photographs of each scratch were taken 24 h after scratching.

### 2.11 Immunohistochemistry

Tissue samples from nine different patients including five HNSC patients, two COAD patients, one BRCA patient and one LIHC patient, who were diagnosed with cancer and treated at The First Affiliated Hospital of Anhui Medical University in 2021, were included in the analysis. This study was approved by the Hospital Review Board of The First Affiliated Hospital of Anhui Medical University (Reference number: PJ2021-14-24). The samples were incubated with rabbit anti-CSTF2 (1:250; bs-14090P, Bioss, China) at 4°C overnight.

### 2.12 Statistical Analysis

The Spearman Correlation test was used to assess the correlation between CSTF2 expression and targets of interest, including immune cell infiltration scores (as described in the previous section for six immune cell types), mismatch repair (MMR) genes and methylation transferase genes. The comparison of CSTF2 expression levels between groups, or between tumor and normal tissues, was performed with paired *t-*tests or the *t-*test, depending on whether the samples are paired or not. Results are considered statistically significant at a *p*-value < 0.05.

## 3 Results

### 3.1 CSTF2 Was Highly Expressed in Endemic Carcinoma

**FIGURE 1:**
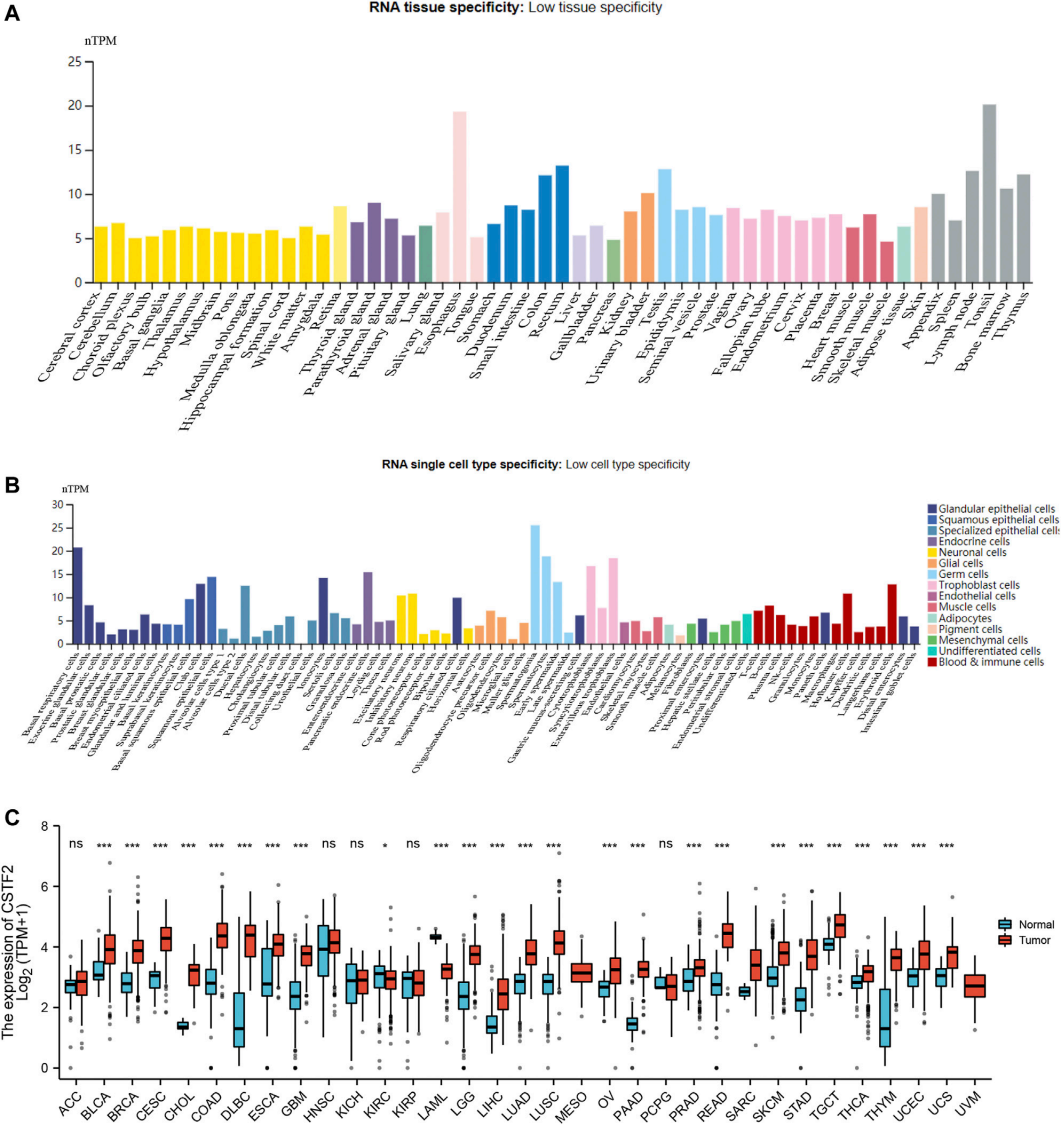
Expression difference of *CSTF2* in pan-carcinoma. **(A)** *CSTF2 mRNA* expression in 31 tissues. **(B)** Expression of *CSTF2* in 21 normal cell lines. **(C)** Differences in expression of *CSTF2* in various tumor cell lines and their corresponding normal tissues. nTPM indicates the normalized expression level of TPM data.

First, we analyzed gene expression levels in each tissue using the HPA database, and we observed gene expression in 31 tissues (Figure 1A). The average TPM value of all individual samples for each human tissue or human cell type was used to estimate the gene expression level. To be able to combine the datasets into consensus transcript expression levels, a pipeline was set up to normalize the data for all samples. For further analysis, we downloaded the data of each tumor cell line from the CCLE database and analyzed the expression levels of 21 tissues according to tissue sources (Figure 1B). Considering the small number of normal samples in TCGA, we combined data from normal tissues in the GTEx database with data from TCGA tumor tissues to analyze the differences in expression of 33 tumors (Figure 1C), and the results showed that CSTF2 was typically overexpressed in tumors compared to normal tissues in the pan-cancer analyses. The above results indicated that CSTF2 may play a key role in the occurrence and development of tumors. Next, we continued to explore the functional mechanism of CSTF2 and its potential value for clinical applications in the future.

### 3.2 *CSTF2* Mutation Did Not Affect the Prognosis of Patients with Tumors

**FIGURE 2:**
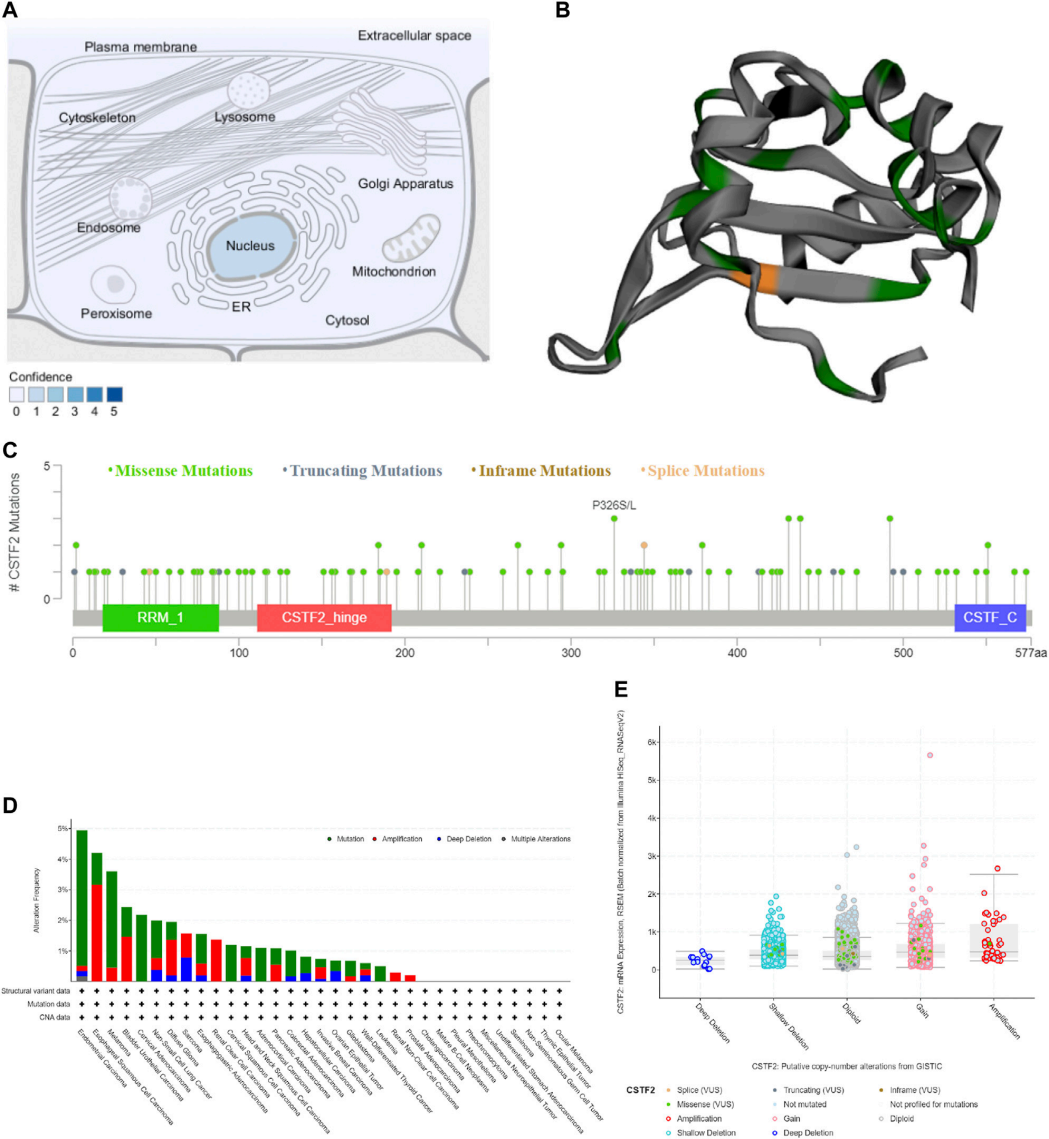
The mutation of *CSTF2*. **(A)** Localization of *CSTF2* in subcellular structures. **(B)** Three-dimensional structure of *CSTF2* protein. Green: missense mutations; Grey: truncating mutations; Orange: inframe mutations. **(C)** Possible sites of mutation. **(D)** Mutations of *CSTF2* in carcinomatous samples. **(E)** Putative copy-number alterations from Genomic Identification of Significant Targets in Cancer (GISTIC) algorithm.

We used the Compartments database to predict the location of CSTF2 in subcellular structures, and the molecule was found in the nucleus (Figure 2A). The three-dimensional structure of CSTF2 suggested the position of a possible mutation of the protein (Figure 2B). Therefore, we predicted CSTF2 mutation sites (Figure 2C), as well as mutations in 10,967 samples (Figures 2D,E). The results showed that the mutation rate of CSTF2 was less than 5% in varieties of carcinoma, and the deep deletion was lower. Through data analysis, we found that the number of mutations found in CSTF2 was low, and the impact of these mutations on patient survival was not statistically significant (Supplementary Figure 1). However, whether CSTF2 mutations play a key role in the development of tumors needs to be further studied.

### 3.3 The Role of CSTF2 in Tumors

**FIGURE 3:**
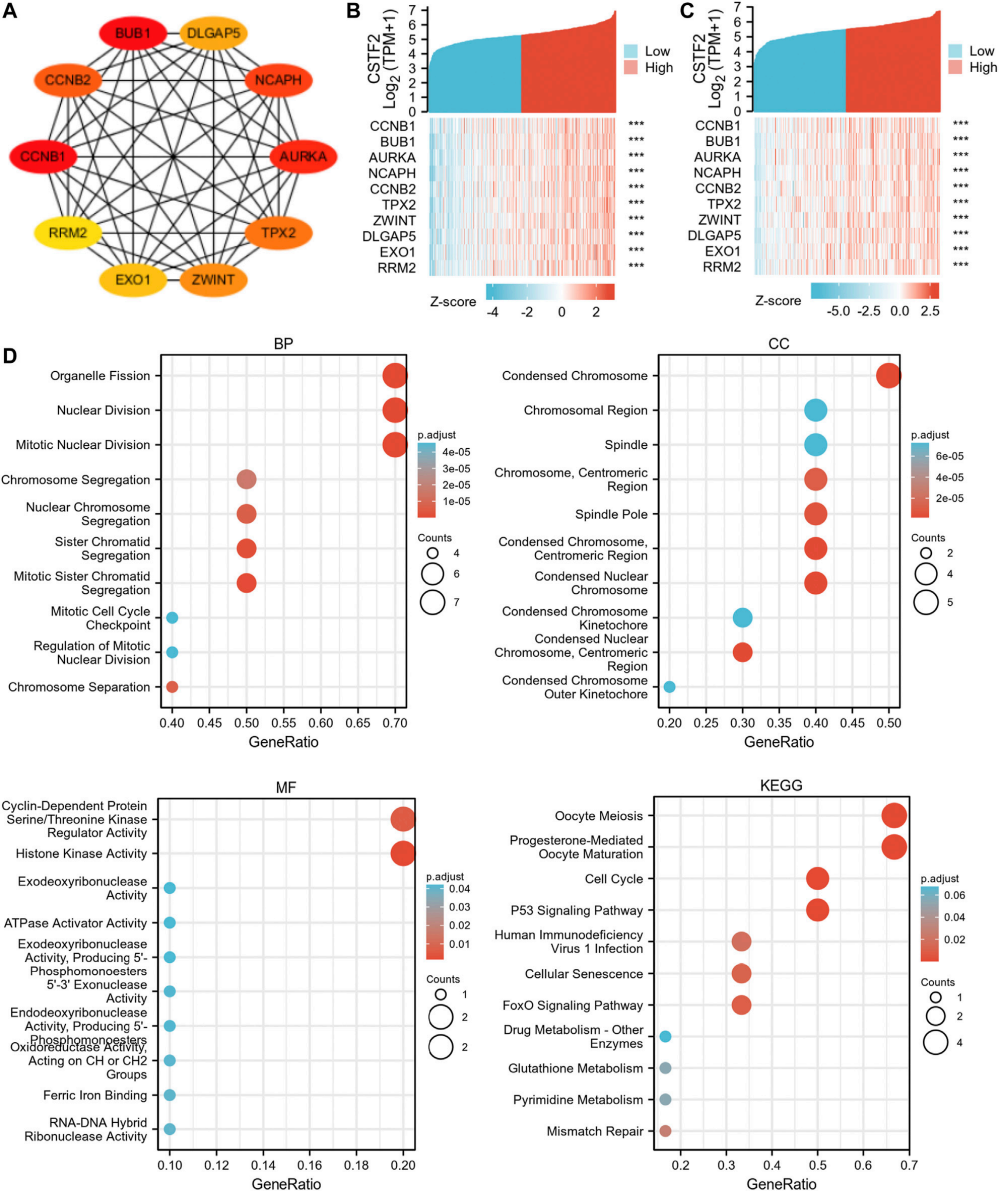
Intracellular function of *CSTF2*. **(A)** Interactive network of hub genes; **(B,C)** Co-expression analysis of hub genes and *CSTF2* in cervical cancer and head and neck cancer. **(D)** GO/KEGG enrichment analysis, and the results were presented in the form of bubble plots.

We used GEPIA database to analyze similar genes of CSTF2 and constructed a protein-protein interaction network using STRING database. Pairing proteins with an interaction score above 0.4 were screened, and information on 1,065 protein interactions was obtained. These protein correlation data were then input into the Cytoscape software, and the top 10 hub genes were obtained by MCC algorithm (Figure 3A). The data sets of cervical cancer and head and neck cancer in TCGA were used for co-expression analysis to verify hub genes (Figures 3B,C). The results showed that 10 hub genes were remarkably associated with CSTF2 overexpression, suggesting that these genes may be closely related to the function of CSTF2. Therefore, we then performed gene ontology (GO) and Kyoto Encyclopedia of Genes and Genomes (KEGG) enrichment analyses (Figure 3D) on these genes, which enhanced nuclear division, histone activity and other functions, and mapped them to chromosome regions. Genes related to cell division, cell cycle, and other signaling pathways were enriched indicating that CSTF2 was likely to affect DNA replication, transcription, and other functions.

**FIGURE 4:**
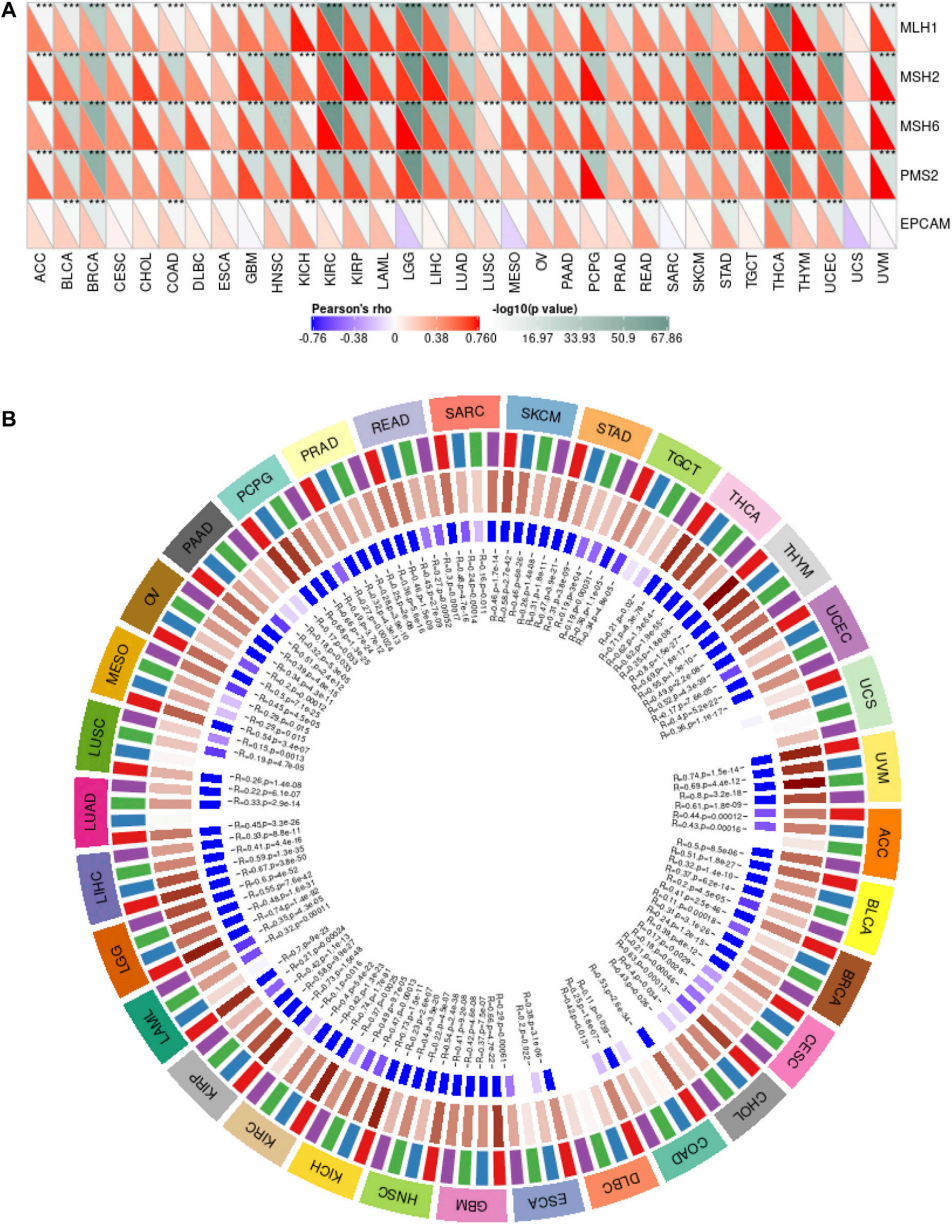
*CSTF2* is involved in DNA mismatch repair and methylation in pan-cancer. **(A)** Correlation between MMR genes and *CSTF2* in endemic cancers. **(B)** Correlation between *CSTF2* expression and methyltransferase in endemic carcinomas (red:DNMT1, blue:DNMT2, green:DNMT3A, purple:DNMT3B).

MMR is an intracellular mismatch repair mechanism. Loss of key genes in this mechanism will result in DNA replication errors that cannot be repaired, leading to the generation of high somatic mutations. Here, we used TCGA expression profile data to evaluate the relationship between mutations and gene expression of five MMR genes: *MLH1*, *MSH2*, *MSH6*, *PMS2*, and *EPCAM* (Figure 4A). The results showed that CSTF2 was highly correlated with MMR-related genes in a variety of tumors, suggesting that CSTF2 may affect the DNA replication mismatch repair process in the nucleus, and thus affecting the tumorigenesis.

GO enrichment analysis showed that histone activity was enhanced, and the most common modification was DNA methylation. DNA methylation is a form of DNA chemical modification that alters genetic expression without altering the DNA sequence. DNA methylation can cause changes in the chromatin structure, DNA conformation, DNA stability and the way DNA interacts with proteins, thus controlling gene expression. DNA methylation is the binding of a methyl group at the 5′-carbon covalent bond of the CpG dinucleotide in the genome under the action of DNA methylation transferase. Therefore, we then analyzed the correlation between the expression of *CSTF2* and the expression of four methyltransferases (red:DNMT1, blue:DNMT2, green:DNMT3A, purple:DNMT3B, Figure 4B). The results showed that CSTF2 may affect DNA transcription through the regulation of methyltransferase.

In order to verify the effect of CSTF2 on DNA mismatch repair and methylation, we knocked down CSTF2 in HN6 cells and observed the mRNA expression of DNA mismatch repair genes and methyltransferase genes (Supplementary Figure 2). The results showed that MSH2, MSH6, and PMS2 increased significantly when CSTF2 was knocked down, indicating that CSTF2 might promote tumorigenesis by increasing DNA mismatch. At the same time, knocking down CSTF2 could also affect the mRNA expression of DNMT1, DNMT2, and DNMT3B. We speculate that this may affect the methylation process of some key genes in cells, resulting in the occurrence and development of tumor. The specific mechanism still needs to be further explored.

### 3.4 CSTF2 Is Associated With Immunoinfiltration in Cancers

**FIGURE 5:**
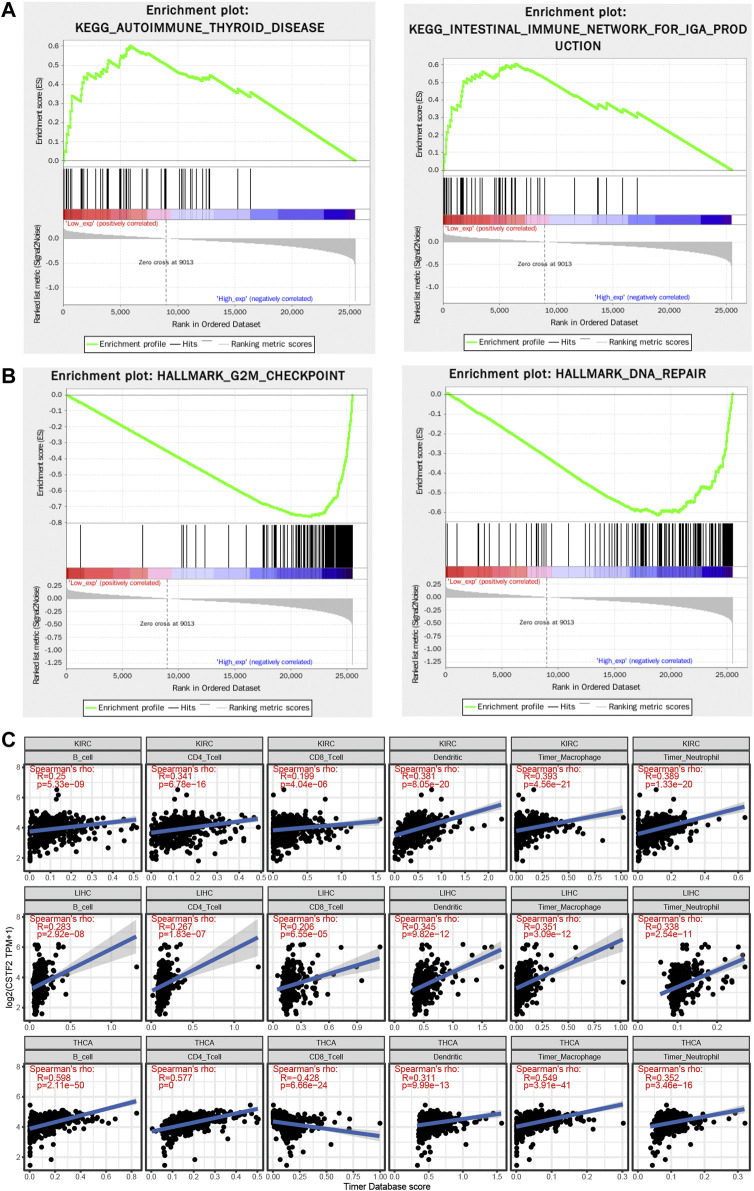
*CSTF2* is associated with immunoinfiltration in the pan-cancer analyses. **(A,B)** Gene set enrichment analysis of *CSTF2* to seek key signaling pathway and hallmark. **(C)** Immunoinfiltration of *CSTF2* in different cancers.

To observe the effect of gene expression on tumor, samples were divided into high and low groups according to gene expression, and the enrichment of KEGG (Figure 5A) and hallmark (Figure 5B) pathways were analyzed by gene set enrichment analysis (GSEA). G2/M checkpoint and DNA repair related pathways were enriched, which was similar to our previous GO enrichment analysis, but immune-related signaling pathway functions were also enriched.

Tumor infiltrating lymphocytes are an independent predictor of sentinel lymph node status and survival in cancer. We investigated whether the expression of CSTF2 is associated with the level of immunoinvasion in different types of cancer. First, we downloaded score data of six immunoinfiltrating cells from the TIMER database for 33 types of cancer. The correlation between CSTF2 gene expression and the scores of these immune cells was analyzed respectively (Figure 5C), and it was found that the expression level of CSTF2 was generally positively correlated with the infiltration of immune cells in tumors. To further analyze the correlation of CSTF2 in different immune cells in various tumors, we used the TISCH database for single-cell data analysis (Supplementary Figure 3). These results suggested that *CSTF2* expression affects the immune infiltration of various tumors and may play a role in regulating tumor microenvironment.

### 3.5 CSTF2 May Be a Potential Anticancer Drug Target

**FIGURE 6:**
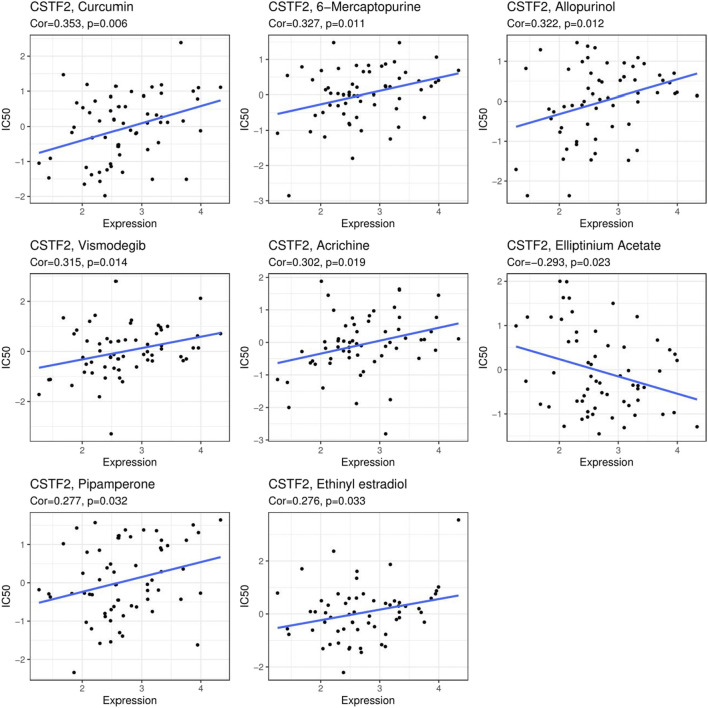
Relationship between *CSTF2* expression and common compound molecule IC_50_.

We analyzed *CSTF2* expression in relation to more than 40 common immune checkpoint genes (Supplementary Figure 4), showing that CSTF2 expression is associated with common immune checkpoints in a variety of cancers, especially in kidney, liver, and thyroid cancers. We further analyzed drug networks in different tumors (Supplementary Table S1) and analyzed the effects of these drug molecules on CSTF2 mRNA or protein (Supplementary Table S2), demonstrating that CSTF2 mRNA or protein could be used as drug targets in a variety of tumors. Next, we analyzed the correlation between CSTF2 expression and the IC_50_ of commonly used antitumor drugs. (Figure 6). The results showed that inhibiting the expression of *CSTF2* could reduce the value of IC_50_ of most antitumor drugs, thereby proving that CSTF2 could be a potential target of anticancer drugs. Consequently, this may provide a new therapeutic approach for oncologists to prescribe personalized treatment plans.

To further clarify the indirect targeting mechanism between CSTF2 and drug components, we submitted the top 10 hub genes obtained by analysis to the DGIdb database (https://dgidb.org/) for matching and finally obtained 68 drug components interacting with these genes. We uploaded interactive information into the Cytoscape software for visual display (Supplementary Figure 5). AURKA primarily matched Aurora A inhibitors such as TAS-119, MK-5108, and SNS-314, which inhibit tumor growth by inducing apoptosis. EXO1 matched FLUOROURACIL and CAPECITABINE, which mainly interfere with cell division and related RNA and protein synthesis, thus blocking the infinite proliferation ability of tumor cells. RRM2 interaction drugs such as HYDROXYUREA, CLOFARABINE, and GEMCITABINE mainly affect the normal division process of tumor cells by blocking DNA synthesis but has no blocking effect on RNA and protein synthesis, while some drugs such as CAPECITABINE can directly promote the apoptosis of tumor cells. SELICICLIB and KENPAULLONE, matched by CCNB1, can selectively inhibit CDK activity and block DNA replication in tumor cells by influencing the cell cycle. These major drug components might function directly or indirectly through CSTF2 *in vivo*, and more evidence is needed to elucidate the regulatory network. Thus, the prediction results of this study provide ideas and directions for future research.

### 3.6 CSTF2 Has Strong Diagnostic Efficacy for a Variety of Cancers

**FIGURE 7:**
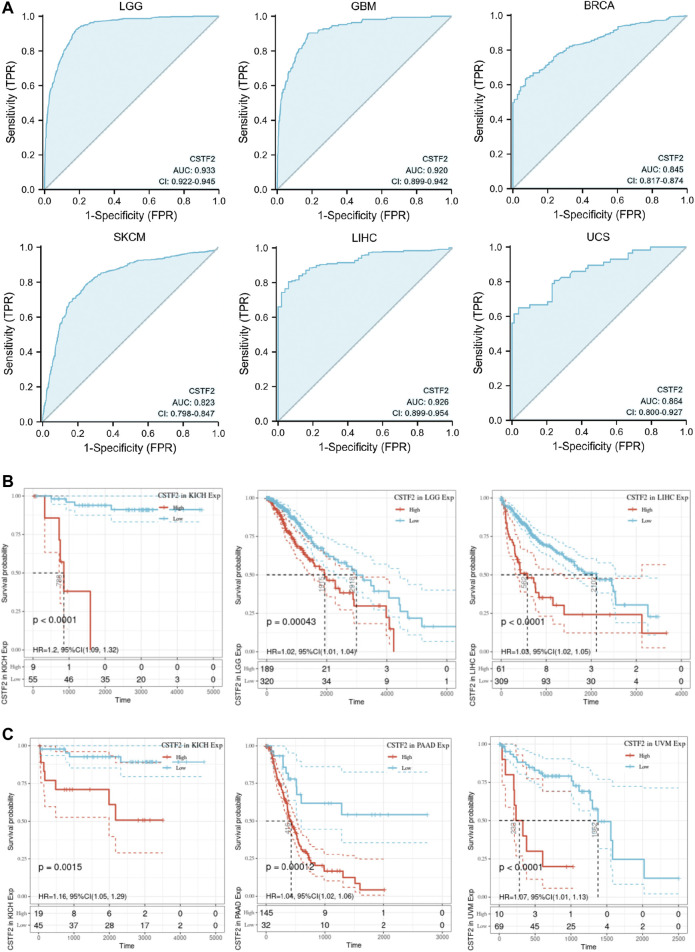
Relationship between *CSTF2* and diagnosis and prognosis of multiple cancers. **(A)** ROC curves of *CSTF2* in multiple cancers. **(B)** Overall survival curve. **(C)** Progression-free survival curve.

We have analyzed the functional role that CSTF2 may play in a variety of cancers and evaluated its potential as an anticancer drug target. We also evaluated the diagnostic efficacy of CSTF2 for tumors. We plotted the ROC curve of CSTF2 in a variety of cancers (Figure 7A), and the results showed that CSTF2 had a strong diagnostic efficacy in a variety of tumors, indicating that the expression of *CSTF2* could distinguish tumors from non-tumors.

We then evaluated the effect of CSTF2 on prognosis in cancer patients. Results of the overall survival curve (Figure 7B) and progression-free survival curve (Figure 7C) showed that CSTF2 had a considerable impact on the prognosis of patients with kidney cancer, glioma, liver cancer, pancreatic cancer, melanoma, and other cancer types (*p*-values < 0.01). These results suggested that CSTF2 could play a role in the diagnosis of tumors and prognosis of cancer patients and has the potential to become a biomarker of several cancers.

### 3.7 The Effect of CSTF2 on Tumors

**FIGURE 8:**
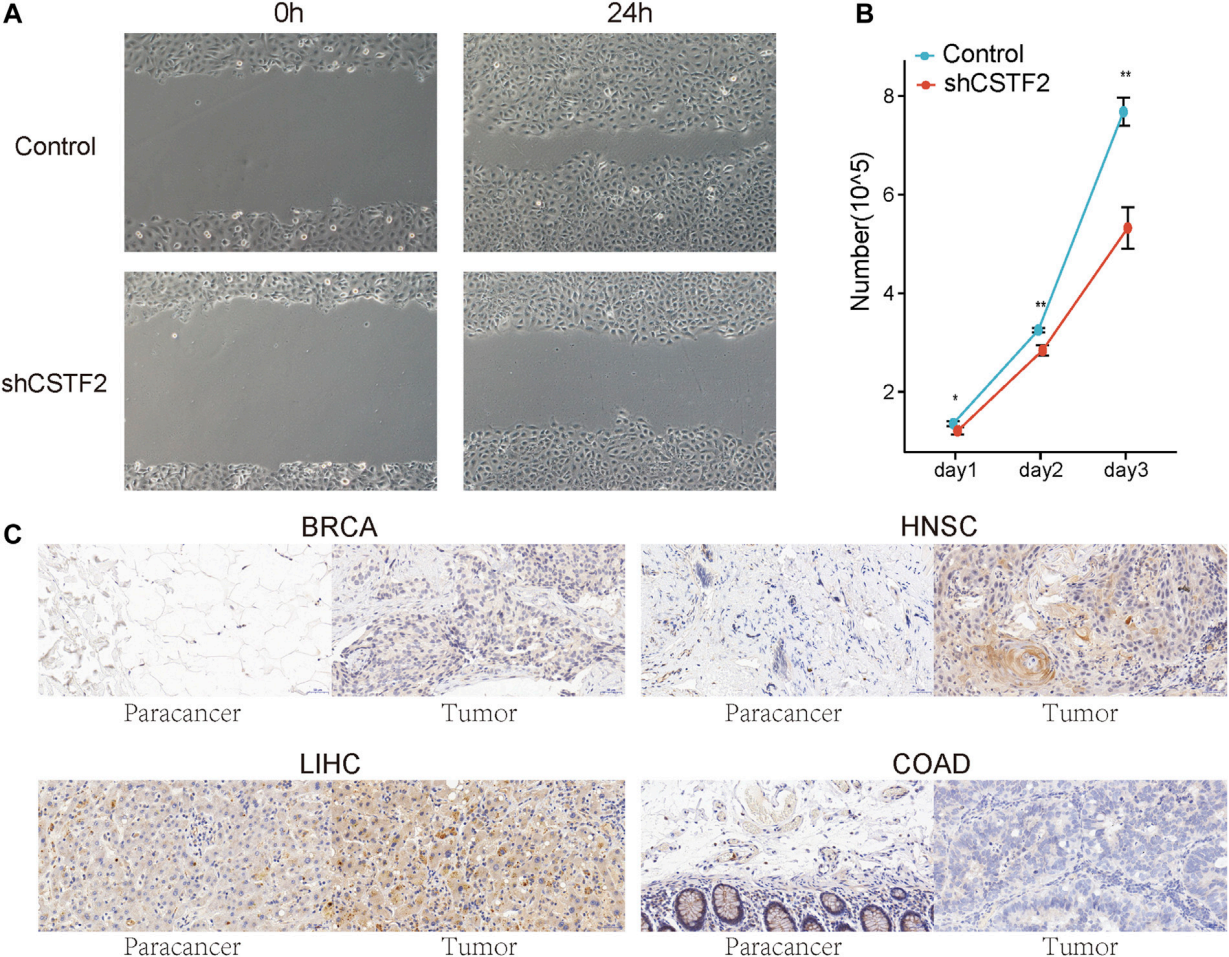
The effect of CSTF2 on tumors. **(A)** The migration of HN6 cells transfected with plko.1/plko.1-shCSTF2 was observed by scratch assay after 24 h. **(B)** The proliferation of HN6 cells with plko.1/plko.1-shCSTF2 was recorded at days 1, 2, and 3. All the experiments were verified by three independent repeated experiments. **(C)** Typical results of normal and tumor tissues in 1 *BRCA* patient, 5 *HNSC* patients, 1 *LIHC* patient, and 2 *COAD* patients.

After knocking down CSTF2 in oral tumor cell lines (Supplementary Figure 6), we observed that cell migration ability was significantly inhibited in oral tumor cell lines compared to that in the control group (Figure 8A). Further, cell proliferation was detected after CSTF2 was knocked down, and inhibition of cell proliferation was observed (Figure 8B). These results suggested that CSTF2 could promote the proliferation of oral tumor cells.

Finally, to verify the results obtained *via* bioinformatics, cancerous and paracancerous tissues were collected from four patients with various tumors for immunohistochemical analysis so as to detect the expression of CSTF2 in the tissues (Figure 8C). The results showed that the expression of CSTF2 in breast invasive carcinoma, head and neck cancer, and liver hepatocellular carcinoma was significantly higher than that in adjacent tissues; however, the difference was not significant in colon adenocarcinoma. This indicated that the mechanism of CSTF2 in regulating tumor *in vivo* may be more complex.

## 4 Discussion

In our study, although the mutation of CSTF2 did not directly affect the prognosis of tumor patients, we speculated that the gene may play an important regulatory role by affecting other important activities in cells. By constructing the protein interaction network of this gene, we found the hub genes and their related functions. The results showed that CSTF2 may influence RNA maturation, and these results are consistent with those of previous reports.

CSTF2 may act as an oncogene to regulate the length of the 3′ non-coding region (UTR) of cancer-related genes in NSCLC (Delfanti et al., 2021). In H460 cells, down-regulation of CSTF2 resulted in an extension of the 3′ UTR of the shortened gene found in tumor tissues of NSCLC, suggesting that CSTF2 may be a therapeutic target for lung cancer (Aragaki et al., 2011).

We also observed that CSTF2 might be involved in DNA repair and methylation. By analyzing the correlation between CSTF2 and typical DNA repair related enzymes and methyltransferases in tumors, we speculated that CSTF2 might affect the expression or activity of key enzymes, thereby affecting the DNA transcription process. This function might affect the expression of several key genes associated with cancer, regardless of the tumor origin, and play an important role in regulating the occurrence and progression of cancer, which was similar to what had been found in previous reports (Harris et al., 2016; Grozdanov et al., 2018b; Gharesouran et al., 2019).

Studies have shown that CSTF2 induces shortening of RAC1 3′-UTR and promotes urothelial carcinoma of the bladder. CSTF2 induces the shortening of RAC1 3′-UTR in umbilical cord blood cells by slowing the AFF1 and AFF4 mediated transcriptional elongation and by interacting with the Gukku motif downstream of RAC1 (Gao et al., 2013). It has been shown that CSTF2 is involved in the tumorigenic function of the shorter RAC1 subtype. Cord blood with both high expression of CSTF2 and short RAC1 subtypes showed a more aggressive disease and poorer prognosis (Chen et al., 2018).

Tumor immunotherapy based on tumor microenvironment is increasingly used in cancer treatment (Tian et al., 2019; Yang et al., 2020; Zhao et al., 2020; Fan et al., 2021). Therefore, we screened the co-expressed gene sets of CSTF2 expression and performed GSEA. The results of GSEA validated our previous conclusions and enriched into immune-related pathways. Although CSTF2 expression was significantly associated with immunoinvasion in a variety of cancers, the results suggested that CSTF2 did not play a decisive role in the tumor microenvironment during the development and progression of cancer. We hypothesized that CSTF2 did not directly affect the response of immune cells to cancer cells but might influence the therapeutic effect of drugs on tumors, and therefore, indirectly affect the prognosis of cancer patients.

Lastly, we analyzed the correlation between CSTF2 expression and effect of drugs. The results suggested that CSTF2 expression was associated with drug compounds in a variety of tumors and may become a direct target for drug action. Our results indicated that CSTF2 could affect the IC50 value of multiple drugs, suggesting that CSTF2 may affect the therapeutic effect of multiple drugs on cancer patients, and thereby affect the prognosis of cancer patients. Our study showed that the expression of CSTF2 could help distinguish a variety of tumors from non-tumors and had good diagnostic efficacy, and CSTF2 was a potential biomarker. In addition to further confirming the regulation of CSTF2 expression in cancer cell function, studies should also focus on the interaction between CSTF2 expression and a variety of drugs in the future to provide new ideas for the treatment of tumor patients.

## Data Availability

The original contributions presented in the study are included in the article/Supplementary Material, further inquiries can be directed to the corresponding authors.

## Glossary

ACC: Adrenocortical carcinoma
BLCA: Bladder urothelial carcinoma
BRCA: Breast invasive carcinoma
CESC: Cervical and endocervical cancers
CHOL: Cholangiocarcinoma
COAD: Colon adenocarcinoma
CTD: Comparative Toxigenomics database
DLBC: Lymphoid Neoplasm Diffuse Large B cell Lymphoma
ESCA: Esophageal carcinoma
FPKM: Fragments Per kilobase Per Million
GBM: Glioblastoma multiforme
GDSC: Genomics of Drug Sensitivity in Cancer
GEPIA: Gene Expression Profiling Interactive Analysis
GO: Gene ontology
GSEA: Gene set enrichment analysis
HNSC: Head and Neck squamous cell carcinoma
KEGG: Kyoto Encyclopedia of Genes and Genomes
KICH: Kidney Chromophobe
KIRC: Kidney renal clear cell carcinoma
KIRP: Kidney renal papillary cell
LAML: Acute Myeloid Leukemia
LGG: Brain Lower Grade Glioma
LIHC: Liver hepatocellular carcinoma
LUAD: Lung adenocarcinoma
LUSC: Lung squamous cell carcinoma
MESO: Mesothelioma
OV: Ovarian serous cystadenocarcinoma
PAAD: Pancreatic adenocarcinoma
PCPG: Pheochromocytoma and Paraganglioma
PRAD: Prostate adenocarcinoma
READ: Rectum adenocarcinoma
SARC: Sarcoma
SKCM: Skin Cutaneous Melanoma
STAD: Stomach adenocarcinoma
TCGA: The Cancer Genome Atlas
TGCT: Testicular Germ Cell Tumors
THCA: Thyroid carcinoma
THYM: Thymoma
TIMER: Tumor Immune Estimation Resource
TISCH: Tumor Immune Single-cell Hub
UCEC: Uterine Corpus Endometrial Carcinoma;
UCS: Uterine Carcinosarcoma
UVM: Uveal Melanoma
