# Supplementary figures and images for: Cleavage Stimulation Factor Subunit 2: Function Across Cancers and Potential Target for Chemotherapeutic Drugs
Source: Front Pharmacol. 2022 Mar 18;13:852469. doi: 10.3389/fphar.2022.852469 (PMC8971630; doi:10.3389/fphar.2022.852469)

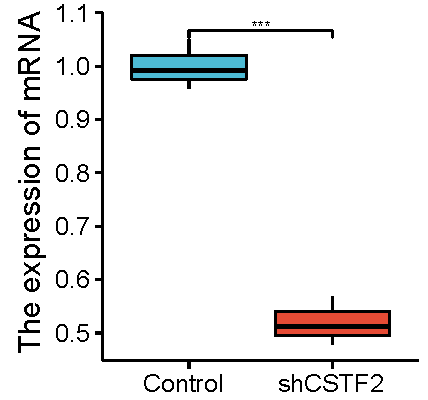

Supplement: Supplementary file 1 [file Image6.TIF]

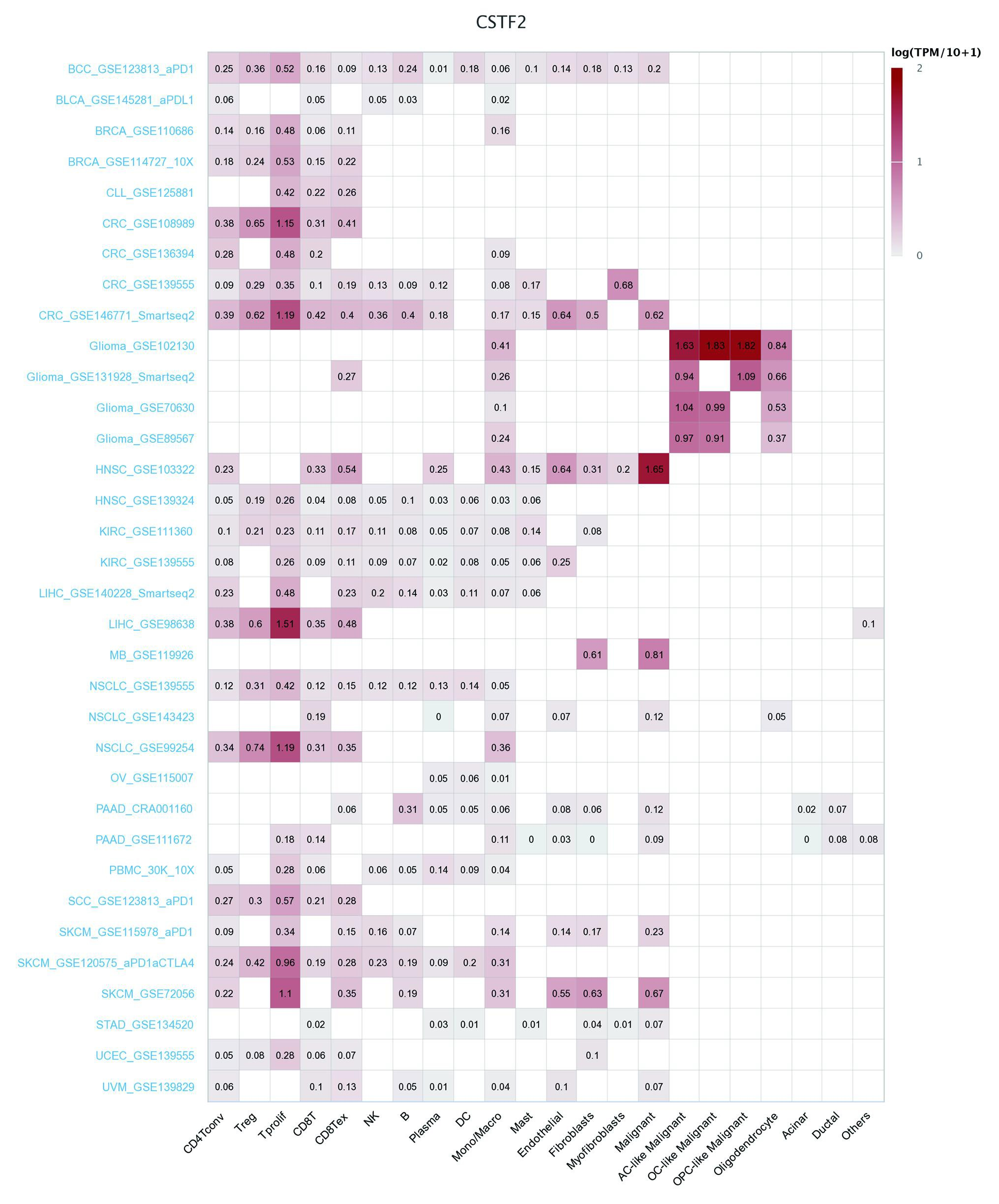

Supplement: Supplementary file 2 [file Image3.TIF]

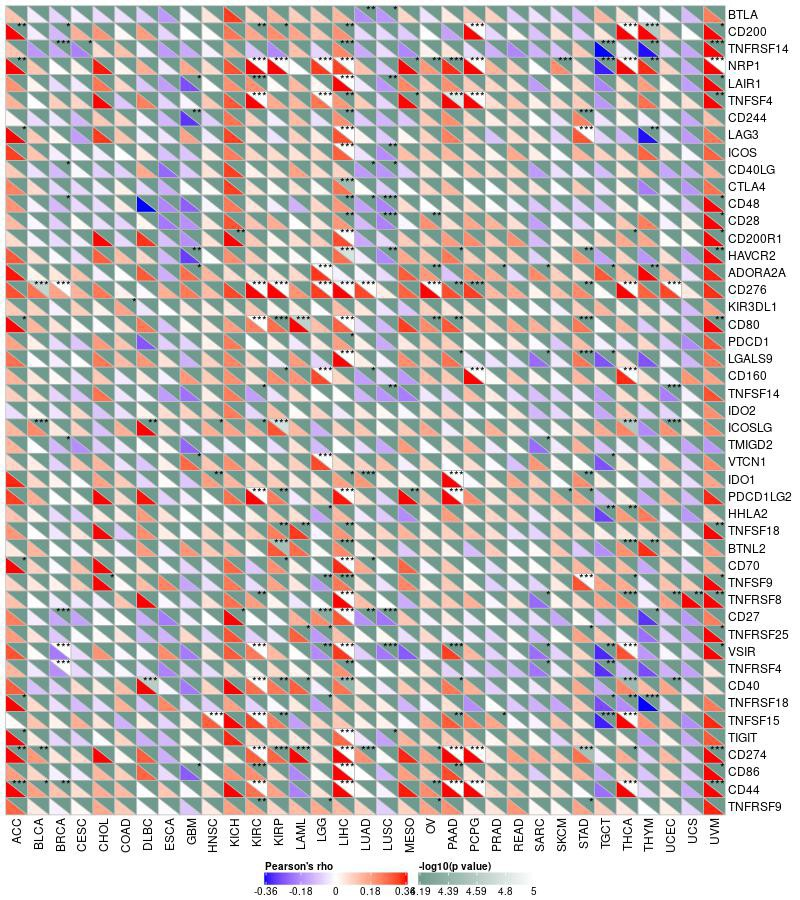

Supplement: Supplementary file 3 [file Image4.TIF]

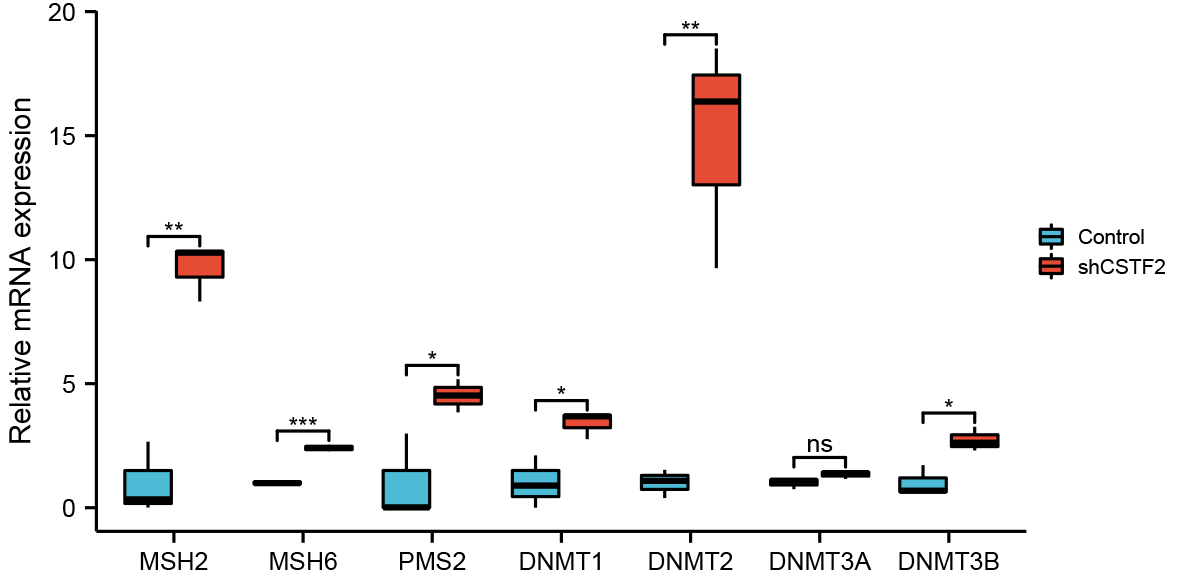

Supplement: Supplementary file 4 [file Image2.TIF]

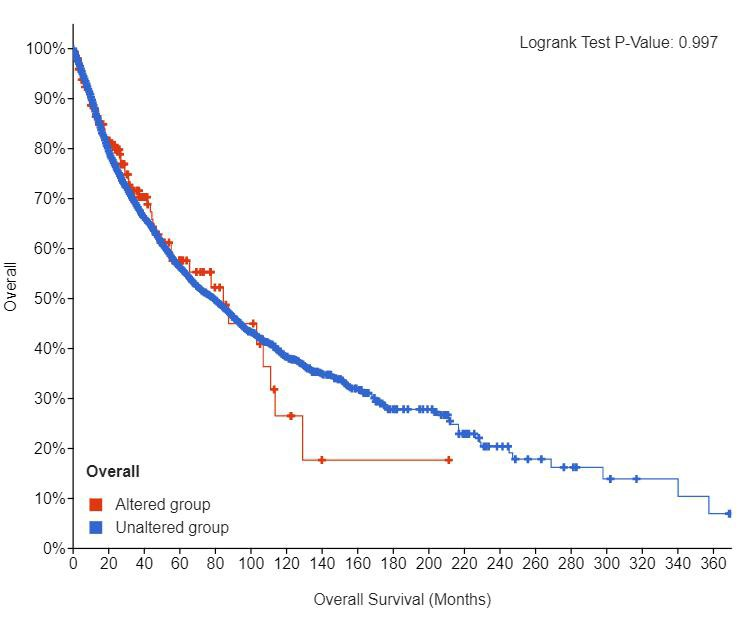

Supplement: Supplementary file 5 [file Image1.TIF]

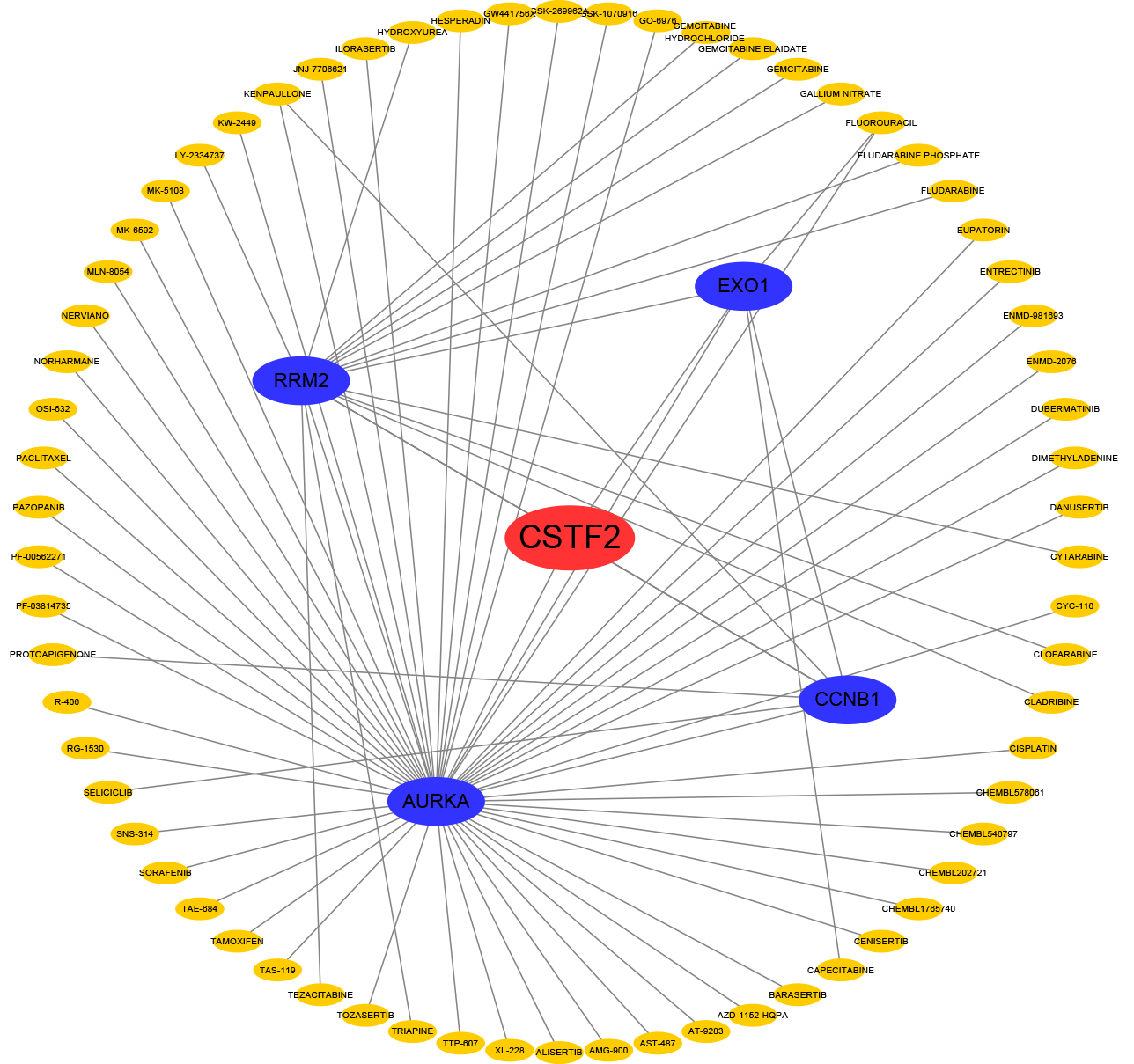

Supplement: Supplementary file 6 [file Image5.TIF]
