# Supplementary material for: Cleavage Stimulation Factor Subunit 2: Function Across Cancers and Potential Target for Chemotherapeutic Drugs
Source: Front Pharmacol. 2022 Mar 18;13:852469. doi: 10.3389/fphar.2022.852469 (PMC8971630; doi:10.3389/fphar.2022.852469)
Supplement: Supplementary file 7 [file DataSheet1.docx]

**Supplementary Tables**

Table S1. CSTF2 may be associated with diseases and targeted drugs

| Disease Name | Inference Network | Inference Score |
| --- | --- | --- |
| Cell Transformation, Neoplastic | Arsenic\|Arsenic Trioxide\|Asbestos, Crocidolite\|Benzo(a)pyrene\|bisphenol A\|chromium hexavalent ion\|Cyclosporine\|Doxorubicin\|Ethanol\|Hydrogen Peroxide\|Plant Extracts\|Quercetin\|sodium arsenate\|Tetradecanoylphorbol Acetate\|Thioacetamide\|Tretinoin\|Vehicle Emissions\|Water Pollutants, Chemical | 66.8 |
| Neoplasm Metastasis | Arsenic\|Arsenic Trioxide\|Benzo(a)pyrene\|bisphenol A\|Carbamazepine\|chromium hexavalent ion\|Dibutyl Phthalate\|Dietary Fats\|Doxorubicin\|Ethanol\|Hydrogen Peroxide\|Plant Extracts\|Quercetin\|Tetrachlorodibenzodioxin\|Tetradecanoylphorbol Acetate\|Tretinoin\|Valproic Acid | 57.65 |
| Neoplasms | Amphetamine\|Arsenic\|Arsenic Trioxide\|Benzo(a)pyrene\|bisphenol A\|chromium hexavalent ion\|Doxorubicin\|Folic Acid\|Petroleum\|Plant Extracts\|Quercetin\|Tetrachlorodibenzodioxin\|Tretinoin\|tris(1,3-dichloro-2-propyl)phosphate\|Valproic Acid\|Vehicle Emissions\|Water Pollutants, Chemical | 56.9 |
| Liver Neoplasms | Acetaminophen\|Arsenic\|Arsenic Trioxide\|Benzo(a)pyrene\|bisphenol A\|Carbamazepine\|Carbon Tetrachloride\|Dietary Fats\|Doxorubicin\|Ethanol\|Ethinyl Estradiol\|Plant Extracts\|Quercetin\|Tetrachlorodibenzodioxin\|Tetradecanoylphorbol Acetate\|Thioacetamide\|Water Pollutants, Chemical | 55.49 |
| Precancerous Conditions | Acetaminophen\|Amphetamine\|Arsenic\|Benzo(a)pyrene\|bisphenol A\|Carbon Tetrachloride\|Cyclosporine\|Dietary Fats\|Ethinyl Estradiol\|Plant Extracts\|Propylthiouracil\|Quercetin\|sodium arsenate\|Tetrachlorodibenzodioxin\|Thioacetamide | 55.07 |
| Neoplasms, Experimental | Arsenic\|Arsenic Trioxide\|Asbestos, Crocidolite\|Benzo(a)pyrene\|bisphenol A\|Doxorubicin\|erucylphospho-N,N,N-trimethylpropylammonium\|Folic Acid\|Plant Extracts\|Quercetin\|Tetrachlorodibenzodioxin\|Tetradecanoylphorbol Acetate\|Thioacetamide\|Tretinoin | 46.21 |
| Lung Neoplasms | Arsenic\|Arsenic Trioxide\|Asbestos, Crocidolite\|Benzo(a)pyrene\|bisphenol A\|Carbon Tetrachloride\|chromium hexavalent ion\|Doxorubicin\|Ethanol\|Plant Extracts\|Propylthiouracil\|Quercetin\|Tetrachlorodibenzodioxin\|Tetradecanoylphorbol Acetate\|Vehicle Emissions | 44.33 |
| Skin Neoplasms | Arsenic\|Benzo(a)pyrene\|Dietary Fats\|Doxorubicin\|Glucose\|Hydrogen Peroxide\|Plant Extracts\|Quercetin\|Tetrachlorodibenzodioxin\|Tetradecanoylphorbol Acetate\|Thapsigargin\|Tretinoin | 44.25 |
| Breast Neoplasms | Arsenic\|Arsenic Trioxide\|Atrazine\|Benzo(a)pyrene\|bisphenol A\|Carbamazepine\|Cyclosporine\|Dietary Fats\|Doxorubicin\|Ethanol\|Folic Acid\|Plant Extracts\|Quercetin\|Tetrachlorodibenzodioxin\|Tretinoin\|Vehicle Emissions\|Water Pollutants, Chemical | 43.91 |
| Liver Neoplasms, Experimental | Acetaminophen\|Arsenic Trioxide\|Benzo(a)pyrene\|bisphenol A\|Carbon Tetrachloride\|Dietary Fats\|Doxorubicin\|Ethanol\|Ethinyl Estradiol\|Plant Extracts\|Quercetin\|Tetrachlorodibenzodioxin\|Thioacetamide | 41.65 |

Table S2. Regulation of common pharmaceutical components on CSTF2

| Chemical Name | Chemical ID | Interaction |
| --- | --- | --- |
| 2',3,3',4',5-pentachloro-4-hydroxybiphenyl | C111118 | 2',3,3',4',5-pentachloro-4-hydroxybiphenyl results in decreased expression of CSTF2 mRNA |
| Acetaminophen | D000082 | Acetaminophen results in increased expression of CSTF2 mRNA |
| Amphetamine | D000661 | Amphetamine results in decreased expression of CSTF2 mRNA |
| Arsenic | D001151 | Arsenic results in increased methylation of CSTF2 promoter |
| Arsenic Trioxide | D000077237 | Arsenic Trioxide results in increased expression of CSTF2 protein |
| Asbestos, Crocidolite | D017638 | Asbestos, Crocidolite results in decreased expression of CSTF2 mRNA |
| Atrazine | D001280 | Atrazine results in increased expression of CSTF2 mRNA |
| AZM551248 | C547126 | AZM551248 results in increased expression of CSTF2 mRNA |
| Benzo(a)pyrene | D001564 | Benzo(a)pyrene affects the methylation of CSTF2 promoter |
| bisphenol A | C006780 | bisphenol A results in decreased expression of CSTF2 mRNA |
| Carbamazepine | D002220 | Carbamazepine affects the expression of CSTF2 mRNA |
| Carbon Tetrachloride | D002251 | Carbon Tetrachloride affects the expression of CSTF2 mRNA |
| chromium hexavalent ion | C074702 | chromium hexavalent ion affects the expression of CSTF2 mRNA |
| Coumestrol | D003375 | Coumestrol results in increased expression of CSTF2 mRNA |
| Cyclosporine | D016572 | Cyclosporine results in decreased expression of CSTF2 mRNA |
| Dibutyl Phthalate | D003993 | Dibutyl Phthalate results in decreased expression of CSTF2 mRNA |
| Diclofenac | D004008 | Diclofenac affects the expression of CSTF2 mRNA |
| Dietary Fats | D004041 | Dietary Fats results in increased expression of CSTF2 mRNA |
| Doxorubicin | D004317 | Doxorubicin affects the expression of CSTF2 mRNA |
| Enzyme Inhibitors | D004791 | [Enzyme Inhibitors results in decreased activity of OGA protein] which results in increased O-linked glycosylation of CSTF2 protein |
| erucylphospho-N,N,N-trimethylpropylammonium | C472787 | erucylphospho-N,N,N-trimethylpropylammonium results in decreased expression of CSTF2 mRNA |
| Ethanol | D000431 | Ethanol affects the splicing of CSTF2 mRNA |
| Ethinyl Estradiol | D004997 | Ethinyl Estradiol results in increased expression of CSTF2 mRNA |
| ethylene dichloride | C024565 | ethylene dichloride results in increased expression of CSTF2 mRNA |
| Folic Acid | D005492 | Folic Acid results in decreased expression of CSTF2 mRNA |
| Glucose | D005947 | [INS protein co-treated with Glucose] results in increased expression of CSTF2 mRNA |
| Hydrogen Peroxide | D006861 | Hydrogen Peroxide affects the expression of CSTF2 mRNA |
| Ionomycin | D015759 | [Tetradecanoylphorbol Acetate co-treated with Ionomycin] results in increased expression of CSTF2 mRNA |
| K 7174 | C410337 | K 7174 results in decreased expression of CSTF2 mRNA |
| manganese chloride | C025340 | manganese chloride results in increased expression of CSTF2 mRNA |
| methyl cellosolve | C005219 | methyl cellosolve results in increased expression of CSTF2 mRNA |
| N-Methyl-3,4-methylenedioxyamphetamine | D018817 | N-Methyl-3,4-methylenedioxyamphetamine results in decreased expression of CSTF2 mRNA |
| oleoresins | C018620 | oleoresins results in decreased expression of CSTF2 mRNA |
| Petroleum | D010578 | Petroleum affects the expression of CSTF2 mRNA |
| Phthalic Acids | D010795 | Phthalic Acids results in increased expression of CSTF2 mRNA |
| Plant Extracts | D010936 | Plant Extracts results in increased expression of CSTF2 mRNA |
| Propylthiouracil | D011441 | Propylthiouracil results in increased expression of CSTF2 mRNA |
| Quercetin | D011794 | Quercetin results in decreased expression of CSTF2 mRNA and protein |
| S-2-pentyl-4-pentynoic hydroxamic acid | C513635 | S-2-pentyl-4-pentynoic hydroxamic acid results in decreased expression of CSTF2 mRNA |
| sodium arsenate | C009277 | sodium arsenate results in increased expression of CSTF2 mRNA |
| Soman | D012999 | Soman results in increased expression of CSTF2 mRNA |
| Tetrachlorodibenzodioxin | D013749 | Tetrachlorodibenzodioxin affects the expression of CSTF2 mRNA |
| Tetradecanoylphorbol Acetate | D013755 | [Tetradecanoylphorbol Acetate co-treated with Ionomycin] results in increased expression of CSTF2 mRNA |
| Thapsigargin | D019284 | Thapsigargin results in decreased expression of CSTF2 mRNA |
| Thioacetamide | D013853 | Thioacetamide results in increased expression of CSTF2 mRNA |
| Tretinoin | D014212 | Tretinoin results in increased expression of CSTF2 protein |
| trimellitic anhydride | C015559 | trimellitic anhydride results in increased expression of CSTF2 mRNA |
| tris(1,3-dichloro-2-propyl)phosphate | C016805 | tris(1,3-dichloro-2-propyl)phosphate results in decreased expression of CSTF2 mRNA |
| Valproic Acid | D014635 | Valproic Acid affects the expression and splicing of CSTF2 mRNA |
| Vehicle Emissions | D001335 | Vehicle Emissions results in decreased methylation of CSTF2 gene |
| Water Pollutants, Chemical | D014874 | Water Pollutants, Chemical affects the expression of CSTF2 mRNA |

**Supplementary Figures**

Supplementary Fig. 1. Survival curve of patients with CSTF2 and KM mutations.

Supplementary Fig. 2. After knocking down CSTF2 in HN6 cells, the expression of MSH2, MSH6, PMS2, DNMT1, DNMT2, DNMT3A and DNMT3B mRNA was detected by quantitative real-time PCR.

Supplementary Fig. 3. Correlation of CSTF2 with various immune cells in the single-cell pan-cancer dataset.

Supplementary Fig. 4. Correlation between CSTF2 and the expression of immune checkpoint genes in pan-cancer.

Supplementary Fig. 5. Interaction between the top 10 hub genes of CSTF2 and matched drugs.

Supplementary Fig. 6. After the specific plko.1/plko.1-shCSTF2 plasmids were transfected into HN6 cells with lipofectamine 3000 (Invitrogen), the expression of CSTF2 mRNA was detected by quantitative real-time PCR.
